# Monitoring HIV Testing in the United States: Consequences of Methodology Changes to National Surveys

**DOI:** 10.1371/journal.pone.0125637

**Published:** 2015-04-30

**Authors:** Michelle M. Van Handel, Bernard M. Branson

**Affiliations:** Division of HIV/AIDS Prevention, National Center for HIV/AIDS, Viral Hepatitis, STD, and TB Prevention, Centers for Disease Control and Prevention, Atlanta, Georgia, United States of America; Centers for Disease Control, TAIWAN

## Abstract

**Objective:**

In 2011, the National Health Interview Survey (NHIS), an in-person household interview, revised the human immunodeficiency virus (HIV) section of the survey and the Behavioral Risk Factor Surveillance System (BRFSS), a telephone-based survey, added cellphone numbers to its sampling frame. We sought to determine how these changes might affect assessment of HIV testing trends.

**Methods:**

We used linear regression with pairwise contrasts with 2003-2013 data from NHIS and BRFSS to compare percentages of persons aged 18-64 years who reported HIV testing in landline versus cellphone-only households before and after 2011, when NHIS revised its in-person questionnaire and BRFSS added cellphone numbers to its telephone-based sample.

**Results:**

In NHIS, the percentage of persons in cellphone-only households increased 13-fold from 2003 to 2013. The percentage ever tested for HIV was 6%–10% higher among persons in cellphone-only than landline households. The percentage ever tested for HIV increased significantly from 40.2% in 2003 to 45.0% in 2010, but was significantly lower in 2011 (40.6%) and 2012 (39.7%). In BRFSS, the percentage ever tested decreased significantly from 45.9% in 2003 to 40.2% in 2010, but increased to 42.9% in 2011 and 43.5% in 2013.

**Conclusions:**

HIV testing estimates were lower after NHIS questionnaire changes but higher after BRFSS methodology changes. Data before and after 2011 are not comparable, complicating assessment of trends.

## Introduction

Estimating the percentage of persons tested for human immunodeficiency virus (HIV) is important for monitoring outcomes of HIV prevention efforts in the United States, adoption of the Centers for Disease Control and Prevention’s (CDC’s) 2006 *Revised Recommendations for HIV Testing of Adults*, *Adolescents*, *and Pregnant Women in Health-Care Settings* [1], and progress toward the goals of the National HIV/AIDS Strategy [2]. For over 15 years, the National Health Interview Survey (NHIS) and the Behavioral Risk Factor Surveillance System (BRFSS) have included questions on HIV testing that served as the basis for numerous analyses of HIV testing [3–7]. However, recent changes to these surveys might affect estimates of HIV testing and the assessment of trends in the percentage of persons who have ever been tested.

NHIS is a nationally representative annual, cross-sectional, multistage probability sample household survey that provides prevalence estimates for a broad range of health measures for the civilian, noninstitutionalized U.S. population. Estimates are based on in-person interviews with a nationally representative sample of adults aged ≥18 years [8]. NHIS includes questions on household telephone numbers to permit recontacting of survey participants [9]. Surveys are subject to order and context effects due to question wording, format, and order [10, 11]. These effects are especially important when tracking trends over time [12]. In 2011, NHIS dropped the acquired immunodeficiency syndrome (AIDS) Knowledge and Attitudes section of the Sample Adult Questionnaire [13]. Until 2011, this section of the survey asked respondents questions on their perceived chances of getting HIV, reported risks for HIV, whether they had ever been tested for HIV, the reason why they had not been tested if they responded “no,” and, if they responded “yes,” the date and venue of the last HIV test. In 2011, only the “have you ever been tested for HIV” question was retained, but it was moved to the Adult Access to Health Care and Utilization section at the end of the Sample Adult Questionnaire [13]. In 2013, the “reason why they had not been tested” follow-up question was added back to the survey [14]. Responses to the “have you ever been tested for HIV” question may be particularly affected by these changes because both the order and context of this question changed. Before 2011, the inclusion of other HIV-related questions might have prompted respondents to recall a prior HIV test; respondents could also change responses to earlier questions after they were asked follow-up questions about HIV testing later in the section.

BRFSS is a state-based, random-digit-dialed (RDD) telephone survey of the civilian, noninstitutionalized adult U.S. population that collects information on preventive health practices and risk behaviors [15]. Until 2011, the BRFSS sampling frame included only landline telephone numbers and used a method called post-stratification to weight survey responses based on demographic characteristics to adjust for non-response and make the sample more representative of the state population. After evidence for the limitations of using only landline telephone numbers for RDD surveys (because of non-coverage bias with increasing cellphone-only usage) accumulated [9, 16, 17], cellphone numbers were added to the BRFSS sampling frame in 2011 and a new weighting method, iterative proportional fitting (“raking”) was also adopted. The telephone numbers in the new sampling frame were screened to select cellphone-only and cellphone-mainly (≥90% of calls received) persons for the sample. In the BRFSS landline sample, one adult is randomly selected from each sampled landline household [16]. In the cellphone-only sample, adults are selected through screening of a sample of cellphone numbers [16]. Raking includes additional variables in the weighting process to account for declining response rates and to make BRFSS estimates more representative [18].

Previous studies have examined the potential bias from not including persons with only cellphones in national-level telephone surveys [9, 16]. A 2006 study by Blumberg et al. found little difference in some health indicators (e.g., obesity, asthma), but the percentage of persons who reported previous HIV testing was statistically significantly lower in landline than in cellphone-only households [9]. At that time, Blumberg concluded that the degree of bias in telephone surveys was small: the small percentage of households that substituted cellphone for landline telephones was expected to exert less bias on surveys than the nonresponse rate for the foreseeable future [9]. Circumstances have since changed. In the BRFSS pilot study including cellphone numbers in the sampling frame, Hu et al. (2011) found that the landline survey significantly underestimated ever being tested for HIV [16]. The pilot study suggested that the substantial increase in the percentage of adults in cellphone-only households, from 4.5% in 2004 to 34% in 2011 [9, 19], might have practical significance for estimates drawn from RDD telephone surveys.

The objectives of this analysis were (1) to assess changes in the percentage of cellphone-only households in the United States from 2003 to 2013; (2) to compare the percentages of persons in cellphone-only and landline households who reported previous HIV testing; and (3) to compare estimates from NHIS and BRFSS the years before and after methodology changes were implemented to assess how these changes might affect estimates and the ability to monitor trends.

## Methods

### Data Sources

NHIS data from 2003 to 2013 were analyzed for household telephone status and the “have you ever been tested for HIV” variable. The final response rate for the NHIS Sample Adult Questionnaire was 74% in 2003 and 61% in 2013 [8]. Additional information on NHIS can be found at: http://www.cdc.gov/nchs/nhis.htm.

BRFSS data from 2003 to 2013 were analyzed using the “have you ever been tested for HIV” variable. BRFSS is sampled and weighted by state; state-level data were then combined to produce annual national estimates. The annual BRFSS response rate, calculated using standards set by the Council of American Survey Research Organizations and the American Association for Public Opinion Research that makes assumptions about eligibility among potential respondents that were not interviewed, was 53% in 2003 and 46% in 2013 [15]. In 2013, BRFSS response rates were 38% for cellphone numbers and 50% for landline numbers [20]. Additional information on BRFSS can be found at: http://www.cdc.gov/brfss/.

### Data Analysis

This analysis used survey data based on three inclusion criteria. First, only records for respondents aged 18–64 years were included, the age group for which BRFSS asked the HIV-related questions. Second, only records from respondents living in the 50 states and the District of Columbia were included in the analysis to make BRFSS comparable with NHIS. Third, only records with “yes” or “no” responses for the “have you ever been tested for HIV” variable were included in the analysis. Records with “unknown” or “refused” responses or missing data (less than 5% and 8% of records annually in NHIS and BRFSS, respectively) were excluded to minimize underestimation. The final sample sizes during 2003–2013 included 235,389 respondents (73.6% of the total) from NHIS and 2,863,317 respondents (66.2% of the total) from BRFSS. SAS version 9.3, which includes statistical programs that account for complex sample designs, was used to produce nationally representative estimates for all analyses.

Household telephone status was calculated from NHIS based on Blumberg et al. (2006 and 2012) [9, 19]. Briefly, households were categorized as cellphone-only if the respondent lived in a household with only a working cellphone. Households were categorized as landline if the respondent lived in a household with a working telephone that was not a cellphone (but may also have a cellphone); and persons with no working phone were defined as respondents living in a household with no working landline or cellphone. Persons in households with no working phone represented less than 2% of respondents in all years (data not shown). Persons in households with missing telephone status were excluded from the analysis; the percentage excluded ranged from 8% in 2005 to 2% in 2013.

We calculated the estimated percentages and 95% confidence intervals (CIs) of persons aged 18–64 years by age, sex, and race/ethnicity for 2003 (before widespread cellphone-only adoption), 2010 (after significant increase in cellphone-only adoption but before survey changes), and 2011 (the year after the survey changes) to document changes in the demographic characteristics of persons in each source. The demographic characteristics of persons by telephone status were also assessed for 2003 and 2013 to compare changes in the demographic characteristics of persons who live in cellphone-only households with persons in landline households.

We then calculated the percentages of persons in cellphone-only households and the percentages who had ever been tested for HIV in cellphone-only and landline households for each year from 2003 to 2013. Changes in household telephone status and in the percentages of persons who had ever been tested for HIV reported in NHIS and BRFSS were calculated for four analysis time periods: (1) 2003 to 2010, to assess overall trends in each survey before the methodological changes; (2) 2010 to 2011, the years immediately before and after the methodological changes in the two surveys; (3) 2011 to 2013 for BRFSS, when the new methodology was consistent; and (4) two intervals for NHIS: 2011 to 2012, when the new methodology was consistent, and 2012 to 2013, when the questionnaire changed again (i.e., follow-up question was restored). Pairwise contrasts for those time points were calculated using cell-mean regression models. A linear regression model with no intercept was used to fit the corresponding cell-means model, and the parameter estimates from the regression were used as the category means in a one-way analysis of variance. The absolute percentage difference and *p* values were reported to indicate significant changes in the percentage of persons in cellphone-only households and in the percentage of persons who had ever been tested for HIV. A *p* value (two-sided) less than 0.05 was considered significant.

## Results

### Characteristics of Persons in NHIS and BRFSS

In 2003, the distribution of persons was similar for NHIS and BRFSS by age group and sex. NHIS estimated a higher percentage of blacks/African Americans (11.6%, 95% CI: 10.8%-12.3%) and a lower percentage of persons of other race/ethnicity (5.1%, 95% CI: 4.7%-5.5%) than BRFSS (10.1%, 95% CI: 9.9%-10.4% and 7.0%, 95% CI: 6.7%-7.2%, respectively). In 2010, NHIS estimated a higher percentage of persons aged 18–24 years (15.7%, 95% CI: 15.0%-16.4%) than BRFSS (12.3%, 95% CI: 12.0–12.7%). NHIS estimated a higher percentage of blacks (12.3%, 95% CI: 11.5%-13.0%) and Hispanics (15.4%, 95% CI: 14.7%-16.1%) than BRFSS (10.4%, 95% CI: 10.2%-10.7% and 14.3%, 95% CI: 14.0%-14.6%, respectively). These differences between the surveys disappeared in 2011 (Table 1).

**Table 1:** Characteristics of persons aged 18–64 years, NHIS and BRFSS, 2003, 2010, and 2011.

	2003						2010						2011					
	NHIS			BRFSS			NHIS			BRFSS			NHIS			BRFSS		
Characteristic	%	95% CI		%	95% CI		%	95% CI		%	95% CI		%	95% CI		%	95% CI	
**Age group (yrs)**																		
18–24	15.7	15.0	16.5	16.0	15.6	16.3	15.7	15.0	16.4	12.3	12.0	12.7	15.7	15.0	16.5	15.6	15.3	15.9
25–34	21.9	21.3	22.6	21.7	21.3	22.1	21.7	21.0	22.4	20.6	20.3	20.9	21.7	21.1	22.4	21.5	21.1	21.8
35–44	24.5	23.8	25.1	24.7	24.4	25.1	20.9	20.3	21.6	25.4	25.1	25.7	20.6	20.1	21.2	21.2	20.9	21.5
45–64	37.9	37.0	38.7	37.6	37.2	38.0	41.6	40.7	42.5	41.7	41.3	42.0	41.9	41.1	42.7	41.7	41.4	42.0
**Sex**																		
Female	51.1	50.3	51.9	50.2	49.8	50.7	50.6	49.7	51.5	50.0	49.7	50.4	50.6	49.9	51.4	49.8	49.4	50.2
Male	48.9	48.1	49.7	49.8	49.3	50.2	49.4	48.5	50.3	50.0	49.6	50.3	49.4	48.6	50.1	50.2	49.8	50.6
**Race/ethnicity**																		
Black	11.6	10.8	12.3	10.1	9.9	10.4	12.3	11.5	13.0	10.4	10.2	10.7	12.1	11.5	12.7	11.8	11.6	12.1
Hispanic	13.7	13.0	14.4	13.7	13.3	14.1	15.4	14.7	16.1	14.3	14.0	14.6	15.6	14.9	16.3	15.5	15.2	15.8
White	69.7	68.7	70.7	69.2	68.7	69.6	65.6	64.5	66.7	67.7	67.3	68.1	65.3	64.3	66.2	65.1	64.8	65.5
Other	5.1	4.7	5.5	7.0	6.7	7.2	6.7	6.2	7.2	7.6	7.4	7.8	7.1	6.6	7.5	7.6	7.4	7.8
**Total**	**100**	–	–	**100**	–	–	**100**	–	–	**100**	–	–	**100**	–	–	**100**	–	–

Note. 95% CI = 95% Confidence Interval; BRFSS = Behavioral Risk Factor Surveillance System; NHIS = National Health Interview Survey

### Changes in the Percentage of Persons in Cellphone-only Households

**Fig 1:**
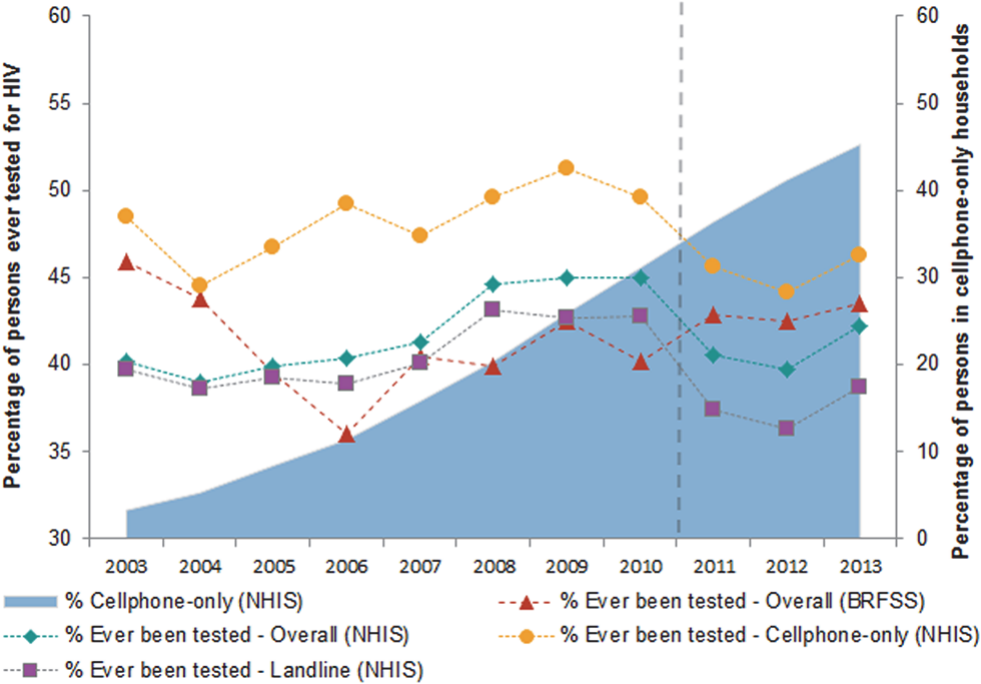
Persons who had ever been tested for HIV and persons in cellphone-only households, 2003–2013. The percentage of persons aged 18–64 years who had ever been tested for HIV and percentage of persons aged 18–64 years in cellphone-only households in the United States was calculated based on NHIS and BRFSS data from 2003 to 2013. The dashed line with a triangle marker indicates the percentage of persons who had ever been tested for HIV based on BRFSS. The dotted line with a diamond marker indicates the percentage of persons who had ever been tested for HIV based on NHIS. The dotted line with a circle marker indicates the percentage of persons in cellphone-only households who had ever been tested for HIV based on NHIS. The dotted line with a square marker indicates the percentage of persons in landline households who had ever been tested for HIV based on NHIS. The area plot indicates the percentage of persons living in cellphone-only households (i.e., are respondents living in a household with only a working cellphone). BRFSS = Behavioral Risk Factor Surveillance System; HIV = human immunodeficiency virus; NHIS = National Health Interview Survey.

In NHIS, the percentage of persons aged 18–64 years in cellphone-only households nationally increased significantly from 3.3% in 2003 to 45.3% in 2013 (42.0% increase, *p* < 0.001). The percentage of persons in landline households showed concomitant decreases (Table 2 and Fig 1). In 2003, the highest percentages of persons in cellphone-only households were persons aged 18–34 years and males (Table 3). In 2013, the highest percentages of persons in cellphone-only households were persons aged 18–34 years, blacks, and Hispanics (Table 3).

**Table 2:** The percentage of persons by household telephone status and percentage of persons tested for HIV by telephone status, NHIS, United States, 2003–2013^a^.

	Telephone status^a^						Ever been tested					
	Cellphone-only^b^			Landline^c^			Cellphone-only^d^			Landline^e^		
Year	%	95% CI		%	95% CI		% tested	95% CI		% tested	95% CI	
2003	3.3	3.0	3.6	95.0	94.6	95.4	48.5	44.6	52.5	39.7	38.8	40.5
2004	5.3	5.0	5.7	93.0	92.5	93.4	44.5	41.4	47.7	38.6	37.8	39.5
2005	8.4	7.9	8.8	90.1	89.6	90.6	46.7	44.3	49.0	39.3	38.4	40.2
2006	11.4	10.6	12.2	87.0	86.2	87.8	49.2	46.6	51.7	38.9	37.8	40.0
2007	15.8	14.9	16.8	83.4	82.4	84.3	47.4	44.8	49.9	40.1	39.0	41.2
2008	20.4	19.3	21.4	78.7	77.6	79.7	49.6	47.4	51.8	43.1	42.1	44.2
2009	25.9	24.8	26.9	73.2	72.2	74.3	51.3	49.4	53.1	42.7	41.6	43.9
2010	31.1	30.0	32.1	67.7	66.7	68.7	49.6	48.1	51.1	42.8	41.7	43.9
2011	36.4	35.5	37.4	62.1	61.1	63.1	45.6	44.3	46.9	37.4	36.4	38.5
2012	41.2	40.1	42.2	57.3	56.3	58.4	44.2	43.0	45.4	36.3	35.1	37.5
2013	45.3	44.3	46.2	52.8	51.9	53.8	46.3	45.1	47.6	38.7	37.5	39.8

Note. 95% CI = 95% Confidence Interval; HIV = human immunodeficiency virus; NHIS = National Health Interview Survey

^a^ Persons in cellphone-only households are respondents living in a household with only a working cellphone. Persons in landline households are respondents living in a household with a working telephone that is not a cellphone. Data not shown for respondents living in a household with no cellphone or landline telephone, which remained relatively stable and <2% of respondents in all years.

^b^ From 2003 to 2013, there was a 42.0% increase in the percentage of persons aged 18–64 years living in cellphone-only households. Linear regression modelling found this change was statistically significant (*p* < 0.001).

^c^ From 2003 to 2013, there was a 42.2% decrease in the percentage of persons aged 18–64 years living in landline households. Linear regression modelling found this change was statistically significant (*p* < 0.001).

^d^ From 2003 to 2010, the percentage of persons in cellphone-only households who had ever been tested remained stable; linear regression modelling found this change was not statistically significant (*p* = 0.6147).

^e^ From 2003 to 2010, there was a 3.1% increase in the percentage of persons in landline households who had ever been tested. Linear regression modelling found this change was statistically significant (*p* < 0.001).

**Table 3:** The percentage of persons aged 18–64 years by household telephone status and select characteristics, NHIS, United States, 2003 and 2013.

	2003						2013					
	Telephone status^a^						Telephone status					
	Cellphone-only			Landline			Cellphone-only			Landline		
Characteristic	%	95% CI		%	95% CI		%	95% CI		%	95% CI	
**Age group (yrs)**												
18–24	6.0	5.1	7.0	91.3	90.3	92.4	56.8	53.9	59.6	41.3	38.4	44.1
25–34	5.8	5.0	6.5	92.2	91.3	93.0	65.1	63.3	66.9	32.1	30.3	33.8
35–44	2.5	2.1	3.0	96.0	95.4	96.5	46.5	44.9	48.2	51.8	50.1	53.5
45–64	1.3	1.0	1.5	97.4	97.1	97.8	30.3	29.2	31.4	68.2	67.1	69.4
**Sex**												
Female	2.9	2.6	3.3	95.7	95.2	96.1	44.6	43.5	45.8	53.5	52.4	54.7
Male	3.7	3.3	4.1	94.3	93.8	94.7	45.9	44.6	47.3	52.1	50.7	53.5
**Race/ethnicity**												
Black	3.3	2.5	4.1	93.3	92.2	94.3	47.5	45.3	49.6	50.7	48.5	52.9
Hispanic	3.4	2.8	4.0	94.0	93.3	94.8	55.1	53.3	56.9	41.9	40.1	43.8
White	3.2	2.9	3.6	95.5	95.1	95.9	42.6	41.4	43.8	55.8	54.5	57.0
Other	4.0	2.6	5.4	94.7	93.1	96.3	42.5	39.8	45.2	55.9	53.2	58.7
**Total**	**3.3**	**3.0**	**3.6**	**95.0**	**94.6**	**95.4**	**45.3**	**44.3**	**46.2**	**52.8**	**51.9**	**53.8**

Note. 95% CI = 95% Confidence Interval; HIV = human immunodeficiency virus; NHIS = National Health Interview Survey

^a^ Persons in cellphone-only households are respondents living in a household with only a working cellphone. Persons in landline households are respondents living in a household with a working telephone that is not a cellphone. Data not shown for respondents living in a household with no cellphone or landline telephone, which remained relatively stable and <2% of respondents in all years.

### Differences in HIV Testing by Household Telephone Status before Survey Changes

In NHIS, the percentage of persons aged 18–64 years who had ever been tested for HIV was consistently higher in cellphone-only households than landline households each year from 2003 (48.5% vs. 39.7%) to 2010 (49.6% vs. 42.8%; Table 2). The percentage of persons in cellphone-only households who had ever been tested remained stable at approximately 49% (1.1% increase, *p* = 0.6147) from 2003 to 2010, but the percentage of persons in landline households who had ever been tested increased significantly from 39.7% in 2003 to 42.8% in 2010 (3.1% increase, *p* < 0.001).

### Trends in HIV Testing in Each Survey before Survey Changes

Trends in the percentage of persons who had ever been tested for HIV differed between NHIS and BRFSS for each time period (Fig 1). In NHIS, the overall percentage of persons ever tested for HIV increased significantly from 40.2% in 2003 to 45.0% in 2010 (4.8% increase, *p* < 0.001; Table 4); in BRFSS, the percentage decreased significantly from 45.9% in 2003 to 40.2% in 2010 (5.7% decrease, *p* < 0.001). However, BRFSS estimates showed significant year-to-year variation in the percentage of persons who had ever been tested during 2007–2010, as indicated by non-overlapping 95% CIs: increases from 2006 to 2007 and 2008 to 2009, and a decrease from 2009 to 2010.

**Table 4:** The percentage of persons tested for HIV, NHIS and BRFSS, United States, 2003–2013.

	NHIS^a^				BRFSS^b^			
Year	N	% tested	95% CI		N	% tested	95% CI	
2003	22,370	40.2	39.4	41.0	188,917	45.9	45.5	46.3
2004	23,102	39.0	38.2	39.8	214,410	43.8	43.4	44.2
2005	22,260	39.9	39.1	40.7	247,169	39.5	39.1	39.9
2006	17,615	40.4	39.4	41.4	241,175	36.0	35.5	36.4
2007	17,098	41.3	40.3	42.3	281,912	40.5	40.1	40.9
2008	15,928	44.6	43.6	45.5	268,596	39.9	39.5	40.3
2009	20,873	45.0	44.1	46.0	265,762	42.5	42.1	42.9
2010	20,375	45.0	44.1	45.9	273,602	40.2	39.9	40.6
2011	24,886	40.6	39.8	41.5	305,792	42.9	42.6	43.3
2012	26,166	39.7	38.8	40.6	290,633	42.5	42.1	42.8
2013	25,701	42.2	41.4	43.1	285,349	43.5	43.2	43.9

Note. N = Unweighted sample size; 95% CI = 95% Confidence Interval; BRFSS = Behavioral Risk Factor Surveillance System; HIV = human immunodeficiency virus; NHIS = National Health Interview Survey

^a^ Linear regression modeling was used to assess for statistically significant changes in the percentage of persons aged 18–64 years ever tested for HIV in NHIS during 2003–2013. From 2003 to 2010, there was a statistically significant 4.8% increase in the percentage ever tested for HIV (*p* < 0.001). From 2010 to 2011, there was a statistically significant 4.4% decrease in the percentage ever tested for HIV (*p* < 0.001). From 2011 to 2012, the percentage ever tested for HIV did not change significantly (*p* = 0.101). From 2012 to 2013, there was a statistically significant 2.5% increase in the percentage ever tested for HIV (*p* < 0.001).

^b^ Linear regression modeling was used to assess for statistically significant changes in the percentage of persons aged 18–64 years ever tested for HIV in BRFSS during 2003–2013. From 2003 to 2010, there was a statistically significant 5.7% decrease in the percentage ever tested for HIV (*p* < 0.001). From 2010 to 2011, there was a statistically significant 2.7% increase in the percentage ever tested for HIV (*p* < 0.001). From 2011 to 2013, there was a statistically significant 0.6% increase in the percentage ever tested for HIV (*p* = 0.016).

### Changes in HIV Testing after Changes to the Surveys

In NHIS, the overall percentage of persons who had ever been tested in 2011, the year the questionnaire changed, was 40.6%, significantly lower than in 2010 (45.0%), a 4.4% decrease (*p* < 0.001). There was no significant change in the percentage ever tested from 40.6% in 2011 to 39.7% in 2012 (*p* = 0.101). In 2013, after the follow-up HIV question was restored, 42.2% had ever been tested, a significant 2.5% increase (*p* < 0.001). When cellphone numbers were added to the sampling frame and the new weighting methodology was implemented in BRFSS in 2011, the percentage ever tested for HIV was significantly higher (42.9%) than in 2010 (40.2%), a 2.7% increase (*p* < 0.001). This increase persisted in 2012 and 2013, with a statistically significant increase to 43.5% in 2013 (*p* = 0.016).

## Discussion

To compare survey data and use them to assess trends, two assumptions must be met: the survey must access representative populations and must measure the same outcomes. In NHIS, the percentage of persons who had ever been tested for HIV increased from 2003 to 2010. In BRFSS, this percentage decreased from 2003 to 2010. However, in BRFSS, as the percentage ever tested for HIV decreased, the population reached by BRFSS changed in a manner that might have exerted a downward bias on the BRFSS estimates. This study found that the number of persons living in cellphone-only households (not sampled by BRFSS) increased substantially between 2003 and 2010, and in cellphone-only households, the percentage of persons who had ever been tested for HIV was consistently higher than in landline households. The populations represented by NHIS and BRFSS became increasingly dissimilar from 2003 to 2010, with lower percentages of younger adults, blacks, and Hispanics in BRFSS than NHIS. When both surveys changed their methodology in 2011, the NHIS demographic estimates remained the same, but BRFSS demographic estimates again resembled those in NHIS. However, the estimates of having ever been tested for HIV were significantly lower in NHIS but significantly higher in BRFSS in 2011 than the estimates from the previous year. These findings suggest that patterns observed before 2011 cannot be compared with estimates during and after 2011. The effect of survey changes will need to continue to be assessed because of continuing changes in NHIS; BRFSS might also consider sampling only via cellphones, because nearly all households are predicted to have a cellphone in the near future [17].

### HIV Testing Estimates before Survey Changes

NHIS HIV testing estimates before 2011 are plausible. NHIS, as an in-person interview, has not been subject to the biases of increasing cellphone substitution for landlines. In addition, NHIS has had relatively high response rates (74% in 2003 and 61% in 2013) [8]. The percentage of persons aged 18–64 years in NHIS who had ever been tested for HIV increased from 40% in 2003 to 45% in 2010.

In comparison, BRFSS might have underestimated HIV testing before 2011. Our findings are similar to previous studies that found that landline-only surveys underestimate the percentages tested for HIV [9, 16]. The use of only landline numbers in the BRFSS sampling frame before 2011 excluded persons who lived in cellphone-only households who were more likely to test for HIV [5, 16, 21]. The poststratification weighting could not account for the expanding cellphone-only population and their higher percentages ever tested, which likely resulted in an underestimation of HIV testing in BRFSS before 2011.

During 2007–2010, BRFSS estimates of the percentage of persons ever tested for HIV showed improbable year-to-year fluctuations. With a cumulative outcome such as having ever been tested for HIV, the percentage would be expected to remain stable or increase over short periods of observation if a consistent population is sampled. The variations in the BRFSS estimates might be due to non-response bias. To better understand the potential for nonresponse bias, the Office of Management and Budget suggests that surveys with response rates less than 80% conduct a nonresponse analysis to assess whether the data are missing at random [22]. BRFSS overall response rates remained below 60% during the analysis time periods. From 2007 to 2010, when the percentage of households that had only a cellphone increased from 16% to 30%, the BRFSS testing estimate more closely resembled the percentage tested in NHIS among persons in landline households. During this time, HIV testing estimates increased in both BRFSS (2.7% increase) and in NHIS for persons in landline households (2.7% increase). This increase in testing among persons in landline households might have mitigated the decreasing trend in the estimate of the percentage ever tested in BRFSS, but still represents an underestimate compared with NHIS.

### HIV Testing Estimates after Changes to the Surveys

Coincident with removing the AIDS Knowledge and Attitudes section of the questionnaire in 2011, NHIS reported a significantly lower percentage of persons who had ever been tested for HIV than during 2007–2010. Similar to 2011, in 2012, an estimated 40% of persons aged 18–64 years had ever been tested for HIV. Before 2011, if respondents answered “no” to the “have you ever been tested for HIV?” question, they were asked the reasons why they had never been tested. In-depth analysis of NHIS response patterns in 2009 and 2010 revealed an average of 6.5% of respondents changed their previous response from “no” to”yes” after being asked why they had not been tested [23]. Without the follow-up question in 2011 and 2012, respondents were not given a second opportunity to recall a previous HIV test. The follow-up question was restored to NHIS in 2013 [14]. The higher percentage ever tested in 2013 (42.2%) compared with 2012 indicates a partial recovery in the estimated level of testing in NHIS, but not to levels observed before 2011 when the HIV test question was asked in the context of other questions about HIV (such as risks for HIV infection). HIV testing will need to continue to be monitored to fully understand the consequences of the changes in context and order of questions in surveys.

When BRFSS added cellphone numbers to the sampling frame and introduced a new weighting methodology in 2011, their estimate of the percentage of persons ever tested for HIV was significantly higher (42.9%) than in 2010 (40.2%). The 2011 BRFSS estimate was also higher than the 2011 NHIS estimate (40.6%), but lower than the 2008–2010 NHIS estimates (~45%). Although the change in the percentage ever tested from 42.9% in 2011 to 43.5% in 2013 was statistically significant, it was only a 0.6% increase, suggesting that the larger 2.7% increase from 2010 to 2011 was more likely due to methodological changes rather than an increase in testing. We found that including cellphone numbers in the 2011 BRFSS sampling frame improved representation of younger persons, blacks, and Hispanics among respondents. Others have also found that including cellphone numbers in BRFSS increased the proportion of interviews conducted with respondents with lower incomes, with lower education levels, and in younger age groups [18]. The new raking methodology to account for non-response incorporates variables such as education, marital status, and telephone ownership in the statistical weighting process to reduce the potential for bias and increase representativeness of estimates [18]. However, further investigation is necessary to determine whether this methodology is sufficient to compensate for the lower (38%) response rate in the cellphone sample and whether a shift to a single-frame cellphone sample is feasible for an RDD telephone survey [17, 20, 24].

### Limitations

This analysis is subject to at least three limitations. First, no ‘gold standard’ exists for estimates of previous HIV testing among persons aged 18 to 64 years. Prior to 2011, NHIS estimates diverged from BRFSS estimates, and we cannot know which is closer to the true percentage of persons tested for HIV. NHIS and BRFSS are based on different sampling methods and survey methods (in-person versus telephone interview) that might produce different HIV testing estimates. Second, estimates of previous HIV testing are based on respondents’ self-report. Some persons might assume they have been tested for HIV when they have not. Others might not report an HIV test or change their response after being asked other questions related to HIV testing. The supplemental NHIS analysis suggests this contextual change might be of sufficient magnitude to reverse conclusions about trends. Lastly, we analyzed only three years of data after the survey changes, and changes to NHIS continue to be made. More years of data after the survey methods stabilize will be needed to evaluate how these changes affect HIV testing estimates.

## Conclusion

The in-person NHIS and telephone-based BRFSS use different methods and produce dissimilar estimates of the percentage of persons who have ever been tested for HIV. Since no ‘gold standard’ exists for estimates of previous HIV testing, careful consideration is needed when selecting data sources for analyses. Before 2011, NHIS estimates were consistent or increased from year to year. In contrast, BRFSS estimates displayed year-to-year variability and improbable short-term decreases in a cumulative outcome that should remain stable or increase. BRFSS estimates were subject to at least two biases: increasing replacement of landlines by cellphones, which were not included in the sampling frame, and response rates below the usual standard for generalizable surveys. In 2011, NHIS changed the location and context of the question regarding previous HIV testing and BRFSS changed its sampling frame to include cellphones and moved to a new method of weighting. After these changes, the NHIS estimate of past HIV testing was significantly lower and the BRFSS estimate, significantly higher. When NHIS restored the follow-up question for HIV testing in 2013, the estimate of HIV testing again increased significantly. Consequently, HIV testing estimates from these surveys, which have been the mainstay for monitoring HIV testing trends, cannot be used to assess trends in HIV testing that include years before and after 2011. While the methodological changes in NHIS and BRFSS continue to be evaluated, we must triangulate from alternate sources to determine the reliability of NHIS and BRFSS estimates after 2011 and to monitor the uptake of HIV testing in the United States.
